# The Sustainability Paradox in Digital Behavior Change Interventions: Narrative Ownership as a Design Principle for Lasting Change

**DOI:** 10.2196/95781

**Published:** 2026-10-15

**Authors:** Tsuyoshi Okuhara, Hiroko Okada, Emi Furukawa

**Affiliations:** 1 Department of Health Communication, School of Public Health, Graduate School of Medicine, The University of Tokyo, 7-3-1 Hongo, Bunkyo-ku, Tokyo, 113-8655, Japan, 81 3-5800-6549; 2 University Hospital Medical Information Network (UMIN) Center, The University of Tokyo Hospital, Tokyo, Japan

**Keywords:** digital behavior change interventions, narrative ownership, sustainability, engagement, habit formation, mobile health, mHealth, digital health, design principles, person-centered care

## Abstract

Digital behavior change interventions (DBCIs), including smartphone apps, wearable devices, and AI-driven adaptive systems, have become a dominant approach for promoting changes in health-related behavior. Although meta-analyses have consistently demonstrated short-term effectiveness, a recurring finding is that these effects are attenuated once user engagement declines or the intervention ends. We describe this phenomenon as the sustainability paradox: the same design features that maximize behavioral success during use, such as automated guidance, real-time feedback, and gamified rewards, may undermine the conditions necessary for the behavior to persist once the intervention is terminated. This viewpoint introduces narrative ownership as a conceptual framework for understanding and addressing this paradox. Narrative ownership refers to the extent to which individuals experience behavior change as an ongoing, interpretable, and self-authored process and comprises 3 components: process framing, narrative repair, and authorship. We argue that many contemporary DBCI architectures unintentionally displace these experiential processes by automating lapse correction, redirecting behaviors without interpretation, and externalizing the attribution of success. We map these tendencies onto 3 structural mechanisms—the erosion of process framing, the foreclosure of narrative repair, and the hollowing of authorship—and distinguish narrative ownership from adjacent constructs such as engagement, self-efficacy, and autonomy support. We also note that narrative ownership is double-edged: the same reflective processes can consolidate negative self-attributions, and its expression varies across cultural contexts, taking a familial or communal rather than strictly individual form in relational settings. Therefore, to translate this framework into practice, we outline 3 design principles for sustainable digital interventions: reflective pauses that support process framing, lapse reinterpretation features that enable narrative repair, and meaning-linking interfaces that strengthen authorship. For each principle, we derive testable propositions—for example, that reflective pauses will improve behavioral maintenance after the intervention ends and that meaning-linking interfaces will sustain behavior after app use is discontinued—along with candidate outcome measures and directions for empirical validation. Together, these principles reposition the user not merely as the recipient of behavioral guidance but also as the author of their own story of change in digital health interventions.

## The Sustainability Paradox

Digital behavior change interventions (DBCIs), including smartphone apps, wearable devices, and AI-driven adaptive systems, have become dominant modalities for promoting health behavior change [1,2]. Meta-analyses and umbrella reviews have confirmed that DBCIs can produce statistically significant short-term improvements in physical activity, dietary behavior, medication adherence, and other health outcomes [3,4]. The proliferation of behavior change techniques (BCTs) embedded in digital platforms [5], combined with advances in personalization, real-time feedback, and evidence-based design frameworks [6,7], has generated considerable optimism regarding the scalability and reach of these interventions.

However, a recurring finding tempers this optimism: the effects of DBCIs tend to attenuate rapidly once the intervention period ends or when user engagement declines [8,9]. The phenomenon of nonuse attrition, in which users progressively disengage from digital interventions, has been recognized as a fundamental challenge since the earliest years of internet-based health interventions [10]. Studies on wearable activity trackers suggest that sustained engagement rarely extends beyond 3 months [11], and a pattern of initial improvement followed by regression toward baseline has been documented across behavioral domains [12]. A recent systematic review found that although external rewards, such as badges and streaks, are the most commonly adopted engagement strategies in DBCIs, their effects lack long-term sustainability [9]. Emerging evidence suggests that even among users who remain actively engaged, behavioral outcomes may still deteriorate; this finding ostensibly indicates a deeper structural issue [13].

The theoretical literature on behavior change has long distinguished between the processes that initiate change and those that sustain it, noting that the determinants of maintenance may differ fundamentally from those of initiation [14]. Theories of motivation, notably self-determination theory (SDT), hold that behavior is more likely to be sustained when it is experienced as autonomous and self-endorsed rather than externally controlled [15]. This viewpoint reframes the sustainability problem in light of this distinction. We propose what we term the sustainability paradox: DBCIs are designed to maximize behavioral success during the period of use, yet the same features that increase short-term success, such as automated guidance, real-time correction, and gamified rewards, may reduce the likelihood that the behavior persists beyond the intervention. This is more than a simple tradeoff between 2 competing design goals; the mechanisms that support short-term behavioral success may also undermine the conditions for long-term persistence. Personalization algorithms anticipate needs before users articulate them; automated feedback corrects lapses before users reflect on them [16]; moreover, gamified reward systems celebrate achievements that users may not experience as their own [17]. The more effectively a DBCI guides behavior, the less likely the resulting change is to be experienced as belonging to the person whose behavior has changed. For example, a weight management app may automatically adjust daily calorie targets after a lapse and suggest a corrective action such as “Take a 10-minute walk to stay on track.” Although this may help maintain short-term adherence, the lapse itself may never become an experience from which the user interprets or learns.

To address this paradox, we introduce narrative ownership (the extent to which individuals experience behavior change as an ongoing, interpretable, and self-authored process) as a conceptual framework and design principle for sustainable DBCIs. We argue that the sustainability paradox can be understood as a structural deficit in narrative ownership and propose 3 design principles that translate this concept into actionable intervention features. We first identify 3 structural mechanisms through which current DBCI architectures may undermine narrative ownership, define their core components, and outline the corresponding design principles, testable propositions, and limitations.

The aim of this viewpoint is to reframe the sustainability of digitally supported behavior change as a problem of *narrative ownership* and to offer a conceptual basis for designing DBCIs that support lasting change. We write for digital health researchers, behavior change intervention designers, and developers of mobile health (mHealth) apps concerned not only with engaging users but also with sustaining behavior after an intervention ends.

This viewpoint makes 4 contributions. First, we advance, as a theoretical proposition, the *sustainability paradox*: the design features that maximize short-term behavioral success may undermine the conditions for long-term persistence. Second, we propose *narrative ownership* as a conceptual framework for understanding this paradox. Third, we map 3 structural mechanisms in current DBCI architectures onto the 3 components of narrative ownership–process framing, narrative repair, and authorship. Finally, we translate the framework into 3 design principles—reflective pauses, lapse reinterpretation features, and meaning-linking interfaces—and outline testable propositions. We further consider the double-edged nature of narrative ownership and its variation across cultural contexts. Throughout, we use the term *guided but unowned behavior* to describe behavior that a system successfully directs but that the user does not experience as their own, and that is therefore vulnerable to abandonment once the system is removed.

## Why Digital Interventions Fade: 3 Structural Mechanisms

### Overview

The dominant explanation for the attenuation of DBCI effects centers on engagement: users stop using the intervention, and behavior change dissipates accordingly [18]. Although engagement is undeniably important, this explanation treats the problem as primarily technical, involving the design of more compelling interfaces and smart notification systems. A recent international consensus study identified a lack of agreed-upon metrics for engagement and evidence on how better engagement improves outcomes as key unresolved challenges [13]. Therefore, we propose that deeper structural issues should be addressed. Even when users remain engaged, the architecture of many DBCIs can systematically undermine the 3 experiential processes critical for sustained behavior change. Importantly, DBCIs are heterogeneous, and not all of them displace user interpretation in the same way; some already incorporate reflective, autonomy-supportive, or meaning-based components. The concern developed here is therefore directed not at automation as such, but at what we term substitutive automation that replaces the user’s own interpretation, as distinct from supportive automation that prompts reflection, offers choices, or helps users recognize patterns [19]. The 3 mechanisms below describe a structural tendency in prevailing designs rather than a uniform property of all DBCIs.

### Automated Guidance and the Erosion of Process Framing

The first mechanism arises from algorithmic systems in contemporary DBCIs that detect behavioral deviations in real time and respond with immediate corrective guidance [16,20]. When a user’s dietary intake deviates from a target, the system recalculates the meal plan, and when step counts fall short, an adaptive prompt suggests a brief walk. This automated responsiveness is efficient but carries an unexamined cost: it removes the temporal space in which users might reflect on why a lapse occurred and situates it within their broader trajectory of change.

Behavior change, particularly in chronic illness self-management, is not experienced as a series of isolated behavioral events but as an unfolding process that includes hesitation, interruption, and renewed effort [21,22]. A recent narrative review of qualitative studies on the self-management of chronic illness highlights how individuals construct meaning by caring for themselves over time, a process that requires narrative continuity [23]. When algorithmic systems process lapses instantaneously, treating them as errors to be corrected rather than experiences to be understood, users lose the opportunity to frame these events as part of an ongoing process. The lapse becomes something the system handles and not something the person lives through. Thus, the capacity to situate individual setbacks within a continuous, personally meaningful trajectory (*process framing*) tends to be eroded by the same efficiency that makes these systems attractive. For instance, a person attempting to establish a daily walking routine may skip several days due to work stress or family obligations. If the system immediately responds with a prompt such as “You’re behind your goal, take a short walk now,” the behavior may resume without the user ever reflecting on why the interruption occurred or how similar situations might be handled in the future.

### Notification-Driven Behavior and the Foreclosure of Narrative Repair

The second mechanism operates through notification-based behavioral prompts. Push notifications are among the most widely used engagement strategies in DBCIs [18,24], and their effectiveness in triggering immediate behavioral responses is well documented [25]. However, when a notification redirects a user from a lapse toward the “correct” behavior, it performs an override rather than a repair. The user moves past the lapse without interpreting it.

*Narrative repair,* defined as the reinterpretation of a lapse within one’s personal narrative such that it becomes a source of learning rather than a fixed mark of failure, requires a moment of meaning-making that notification-driven redirection tends to foreclose [26,27]. In the relapse prevention literature, the capacity to reinterpret a lapse as a correctable event rather than as evidence of personal inadequacy is recognized as a critical determinant of whether a lapse escalates into full relapse [28,29]. However, most DBCI architectures bypass this interpretation process. For example, someone trying to reduce alcohol consumption may interpret an episode of heavy drinking as either proof of personal failure or a signal of a stressful context that requires new coping strategies. Narrative repair refers to the latter process of reinterpretation. In many DBCIs, however, this interpretive moment is short-circuited: lapses are not narratively repaired but are algorithmically overwritten. Consequently, the experiential residue of lapses, including self-blame, confusion, and demoralization, may accumulate beneath the surface of apparently successful behavioral redirection.

### Externalized Success Attribution and the Hollowing of Authorship

The third mechanism concerns how success is experienced and to whom or what it is attributed. Gamification elements, such as streaks, badges, leaderboards, and progress visualizations, are widely incorporated into DBCIs to sustain motivation [17,30]. Gamification is not inherently problematic; well-designed reward systems can support competence, autonomy, and meaning [31]. Our concern is narrower, focusing on reward structures that supply the interpretation of success rather than supporting the user’s own. These features not only effectively celebrate behavioral achievements but also shape the attributional structure of success in ways that may undermine the user’s sense of *authorship*.

Consider the experiential sequence: when a user reaches a step count target, the app announces the achievement, frames it within its visual language of progress bars and congratulatory messages, and delivers the reward through its incentive system. The interpretive framework through which success is understood (what it means, why it matters, and how it connects to the user’s life) is supplied by the system rather than constructed by the user. Research on personal informatics systems has shown that the manner in which data are collected, visualized, and reflected back to users fundamentally shapes their self-understanding [32,33]. Over repeated cycles, this externalization of the interpretive framework may prevent behavioral achievements from being integrated into the user’s self-understanding. The achievement remains legible within the app’s logic but not within the user’s own narrative of who they are becoming [34].

This concern resonates with long-standing critiques of nudge-based interventions, which have been noted to risk relocating agency from the individual to the choice architect [35,36]. This does not suggest that external feedback is inherently detrimental; it becomes problematic when it substitutes for, rather than supports, a user’s own interpretation of success. In the context of DBCIs, the issue is not that users are coerced, but that the felt sense of success, the experience of having personally achieved something, is displaced onto the system. Users may meet their behavioral targets while experiencing achievement as something that happened to them rather than something they authored. This state, behavioral success without experiential ownership, constitutes what might be called brittle success: sustainable only as long as external scaffolding remains in place [8]. When the app is deleted, the notification stream ceases, or the device is set aside, the behavior that was never owned may be readily abandoned.

Together, the 3 mechanisms (the erosion of process framing, the foreclosure of narrative repair, and the hollowing of authorship) describe not a failure of technology, but a structural pattern in which the experiential conditions for lasting behavior change are systematically displaced by the same features designed to promote it.

## Narrative Ownership: A Conceptual Framework for Sustainable Design

The 3 mechanisms described previously share a common structure: each mechanism represents the displacement of an experiential process necessary for behavior change to be sustained beyond the presence of external support. We propose that these 3 processes be unified under a single conceptual framework: narrative ownership.

### Definition and Components

As mentioned previously, narrative ownership refers to the extent to which individuals experience behavior change as an ongoing, interpretable, and self-authored process. More precisely, narrative ownership is defined as a state in which the experience of behavior change, including lapses and interruptions, is framed as part of an ongoing process (process framing), remains repairable through reinterpretation within one’s personal narrative (narrative repair), and is held with a sense of authorship as one’s own story (authorship).

The concept of narrative ownership draws upon several strands of narrative research. Narrative identity research explains how individuals understand themselves through the stories they construct about their lives [37], whereas work on narrative coherence highlights the importance of temporal continuity and interpretability in personal narratives [38]. In the health context, the illness narrative tradition illustrates how experiences of disruption and recovery are interpreted and reconstructed over time [26,27].

Narrative ownership, in contrast, is narrower in scope and more specific in application; it targets how a behavior change trajectory, including its lapses, is experienced and interpreted by the individual and asks whether that trajectory is held as the individual’s own ongoing process. It is not a theory of identity or a description of illness experience, but an evaluative concept designed to assess whether the conditions for sustainable behavior change are present in a given intervention context. Narrative ownership comprises 3 components.

*Process framing* is the capacity to situate individual lapses and setbacks within a continuous trajectory that includes the surrounding context that triggers them and what is learned from them. When process framing is operative, a lapse is comprehensible not as an isolated failure, but as a moment within an unfolding experience. *Narrative repair* is the reinterpretation of lapse episodes within one’s personal narrative such that they are reconnected to renewed effort rather than fixed as irreversible failures. *Authorship* is the attribution of change, not as an externally imposed result, but as one’s own story, one that can be told in one’s own words.

These 3 components are conceptually distinct but functionally interdependent. Process framing provides the temporal structure within which narrative repair can occur. Narrative repair, in turn, sustains a sense of authorship that makes continued engagement meaningful rather than merely compliant.

Three points clarify the status of the concept. First, narrative ownership is a user-level psychological construct: a state, realized through the aforementioned 3 processes, rather than an intervention feature. We therefore distinguish narrative ownership as a property of the user’s experience from narrative ownership–supportive design as an intervention-level set of features intended to cultivate that state; a DBCI does not contain narrative ownership but can create conditions that make it more or less likely. Second, the construct is design-sensitive: unlike a stable trait, it can be strengthened or eroded by the architecture of the intervention, which is precisely what makes it useful as an evaluative lens for design. Finally, authorship need not be strictly individual. Although the language of personal narrative may suggest an individualistic frame, in relational and collectivist contexts the relevant narrative may be familial, communal, or shared [39], and authorship may be exercised jointly with family members, care partners, or community networks. Recognizing these relational forms at the level of conceptual development, rather than treating them only as a caveat, extends the construct rather than weakening it.

### Why a New Concept Is Needed

Several established concepts in the DBCI literature address related but distinct phenomena.

*Engagement* has become the central construct in DBCI research; however, its operationalization has been predominantly behavioral and has been measured through use frequency, session duration, and feature use [1,18]. Yardley et al [1] have argued for a distinction between mere interaction with a DBCI and “effective engagement,” which involves cognitive and emotional investment. Engagement captures the user’s interaction with the system but does not capture whether the resulting change is experienced as personally owned.

*Self-efficacy*, defined as the belief in one’s capacity to execute a behavior [40], is a well-established predictor of behavior change, and many DBCIs have been explicitly designed to enhance it [41]. Self-efficacy captures capability beliefs but does not capture meaning-making, through which change experiences are integrated into one’s self-understanding.

*Autonomy support*, derived from SDT [15], emphasizes the provision of choice and minimization of external control. A meta-analysis of SDT-informed health interventions confirmed the importance of autonomy-supportive contexts in sustained behavior change [42]. Autonomy support captures the motivational climate in which behavior is initiated but does not address the interpretive process through which a lapse is either fixed as a failure or repaired within one’s narrative.

The sustainability problem then falls into the gap between the existing constructs. Engagement describes interactions without ownership, self-efficacy describes capabilities without meaning, and autonomy support describes motivational quality without postlapse repair. Narrative ownership is needed because the sustainability problem concerns not only whether users continue to act but also whether they can experience and reinterpret change as their own.

This positioning also clarifies the relationship between engagement and narrative ownership, which are related but nonidentical. Engagement, at least as it is usually operationalized, is largely a matter of quantity—how much and how often a user interacts—although effective engagement also involves cognitive and emotional investment [1]; narrative ownership, by contrast, concerns the quality of that interaction: whether the resulting change is experienced as one’s own. The two can dissociate. A user may be highly engaged yet experience little ownership, as when behavior is sustained by notifications and rewards but never interpreted, a state we have termed guided but unowned behavior; conversely, ownership may persist after active engagement with the app has ended, which is precisely the outcome of interest for sustainability. Narrative ownership is therefore closer to what has been described as effective engagement than to frequency of use, and it complements rather than duplicates adjacent constructs: it differs from self-regulation, which concerns the monitoring and adjustment of behavior toward a goal [43], and from reflective motivation, which concerns deliberative evaluations and intentions [44], in that it concerns how the unfolding experience of change—including its lapses—is interpreted and held as one’s own.

Positioning narrative ownership against existing accounts of maintenance further clarifies its contribution. Theoretical reviews distinguish the determinants of initiation from those of maintenance and propose mechanisms such as maintenance self-efficacy, satisfaction with outcomes, habit formation [45], and self-regulation [8]. Identity-based accounts hold that behavior persists when it is congruent with how people see themselves [46], and relapse prevention models emphasize the interpretation of lapses [28]. Narrative ownership is not an alternative to these accounts but a complement that specifies an experiential precondition they tend to leave implicit: for a behavior to become habitual, identity-congruent, or self-regulated once external support is withdrawn, the trajectory of change—including its lapses—must first be experienced as the person’s own. In this sense, narrative ownership addresses the interpretive layer through which these maintenance mechanisms can take hold.

### Reinterpreting the Sustainability Paradox

The 3 structural mechanisms identified in the previous section can be reinterpreted as a systematic absence of the 3 components of narrative ownership. The erosion of process framing through automated guidance reflects the loss of process framing, which is the first component of narrative ownership. The foreclosure of narrative repair through notification-driven redirection undermines narrative repair, which is the second component. The hollowing of authorship through externalized success attribution weakens authorship, which is the third component. Thus, the sustainability paradox is not merely a problem of declining engagement but a problem of absent ownership. Addressing this issue requires design principles that protect and cultivate narrative ownership.

### The Double-Edged Nature of Narrative Ownership

The same interpretive processes that make narrative ownership adaptive can also operate in the opposite direction. Reflecting on a lapse may help a user reframe it as a surmountable step, but it may equally consolidate a negative self-attribution—“I always fail at this”—because positive and negative self-narratives are built through the same mechanisms [47,48]. Narrative ownership is therefore not inherently benign; cultivating it without regard to content risks reinforcing the very self-blame it aims to dissolve. This carries a direct design implication: features that open space for reflection should also be able to detect markers of negative self-framing and, where appropriate, gently challenge rather than merely record them. Because behavior change is often also an attempt to change something about oneself, a narrative ownership–informed DBCI should be designed to support adaptive, rather than corrosive, self-interpretation.

## Design Principles: Embedding Narrative Ownership in Digital Interventions

### Overview

If the sustainability paradox arises from a structural deficit in narrative ownership, then addressing it requires design principles that actively protect and cultivate narrative ownership within DBCIs. The following principles are not prescriptions for specific interface features, but conceptual guidelines that can be instantiated in diverse ways depending on the behavioral domain, target population, and technological platform [49]. They are also distinct from established BCTs: although self-monitoring, action planning, coping planning, values clarification, and relapse prevention exercises [5] primarily structure what users do, these principles structure how users interpret what happens to them, including their lapses. Contemporary DBCI designs have largely pursued frictionless interactions, minimizing the cognitive effort required of users at each step. The principles proposed here represent a deliberate counterpoint: they introduce what might be called “good friction”: minimal, intentional interruption that supports reflection and self-interpretation without creating unnecessary burden, with the aim of promoting long-term ownership rather than short-term ease [50]. These principles complement rather than replace the technological efficiency of current DBCIs.

### Design for Process Framing: Reflective Pauses

The first principle targets the erosion of process framing. Rather than responding to every detected lapse with immediate algorithmic adjustments, DBCIs can incorporate brief reflective pauses, which are moments during which users are invited to interpret the lapse before receiving corrective guidance. A reflective pause might take the form of a short prompt: “Your activity was lower than usual today. Before we adjust your plan, would you take a moment to reflect on what happened?”

The critical design feature is not the specific wording but the temporal space it creates: a gap between the lapse event and the system’s corrective response, in which the user can situate the experience within their own trajectory of change.

This principle draws on the concept of effective engagement [1], which emphasizes that meaningful interactions involve cognitive and emotional processing and not merely behavioral compliance. It also resonates with calls to reconceptualize just-in-time adaptive interventions (JITAIs) [16,20] in ways that balance responsiveness with opportunities for user reflection. An important caveat is warranted: reflective pauses introduce an additional user burden, and the design challenge is to make reflection brief, optional, and contextually sensitive, while making it available as an invitation rather than imposing it as a requirement [51]. Adaptive systems can learn when a user is receptive to reflection [52] and offer selective pauses.

### Design for Narrative Repair: Lapse Reinterpretation Features

The second principle addresses the foreclosure of narrative repair. Rather than moving users past their lapses as quickly as possible, DBCIs can incorporate features that support the reinterpretation of lapses as learning opportunities. Postlapse prompts such as “Looking back at what happened, what would you like to do differently next time?” invite users to construct a narrative connection between a lapse and its future. Over time, the accumulation of such reflections creates what might be called a learning timeline: a record organized not as a list of failures, but as a chronological narrative of challenges encountered and lessons derived.

This principle is informed by the relapse prevention literature, in which the reinterpretation of lapses as correctable events is recognized as critical for preventing escalation to full relapse [28,29]. It is also connected to qualitative research showing that individuals who redefine relapse as part of a recovered social identity are more likely to achieve sustained abstinence [53]. In the digital context, lapse reinterpretation features translate these clinical insights into scalable design elements.

### Design for Authorship: Meaning-Linking Interfaces

The third principle targets the hollowing of authorship. To cultivate authorship, DBCIs can incorporate meaning-linking interface design elements that explicitly connect behavioral goals to users’ self-defined values. At goal setting, users might articulate why a particular target matters to them: “What makes this goal important in your life right now?”

The resulting statements are then displayed alongside quantitative progress indicators to ensure that numerical achievements are continuously linked to their narrative meaning. For example, a step count display may be accompanied by the user’s own words: “I want to be able to play with my grandchildren without getting out of breath.”

This juxtaposition of metrics and meanings anchors the experience of success in the user’s authorship rather than in the app’s reward architecture.

This principle resonates with value-based approaches in health behavior research [54] and with the capabilities approach to person-centered care [55]. It also shares conceptual grounding with the value clarification process in acceptance and commitment therapy, which helps individuals identify and commit to personally meaningful directions for action [56]. The key distinction is that meaning-linking interfaces do not automate this process but embed it within the user’s own narrative, ensuring that values remain personally articulated rather than system-generated.

### Summary: From 3 Mechanisms to 3 Design Principles

Table 1 summarizes the correspondence among the 3 components of narrative ownership, the structural mechanisms that undermine them, and the design principles proposed to address them.

**Table 1.** Mapping structural mechanisms to narrative ownership components and corresponding design principles.

Components of narrative ownership	Structural mechanisms	Design principles	Illustrative features	Hypothesized outcomes
Process framing	Erosion through automated corrective guidance	Reflective pauses	Postlapse reflection prompt before algorithmic adjustment Across domains: pause to reflect before the app readjusts a calorie target (diet), a missed-dose check-in (medication adherence), or a rescheduled session after skipped activity (physical activity)	Better learning from lapses and improved behavioral maintenance after the intervention ends
Narrative repair	Foreclosure through notification-driven redirection	Lapse reinterpretation features	Learning timeline and postlapse meaning-making prompts Across domains: reframing a heavy drinking episode as a stress signal (alcohol reduction) or a smoking slip as a cue to revise coping plans (smoking cessation)	Reduced self-stigma after lapses and faster re-engagement with behavioral targets
Authorship	Hollowing through externalized success attribution	Meaning-linking interfaces	User-defined value statements displayed alongside behavioral metrics Across domains: pairing step counts (physical activity) or glucose logs (diabetes self-management) with the user’s own stated reason for the goal	Greater behavioral persistence after the app is discontinued

Figure 1 illustrates the contrast between a conventional DBCI design pathway in which lapses are algorithmically corrected without user interpretation (Figure 1A) and a narrative ownership–informed pathway in which each design principle activates a component of narrative ownership (process framing, narrative repair, or authorship), which in turn supports a hypothesized effect that contributes to behavioral maintenance after the intervention ends (Figure 1B).

**Figure 1.**
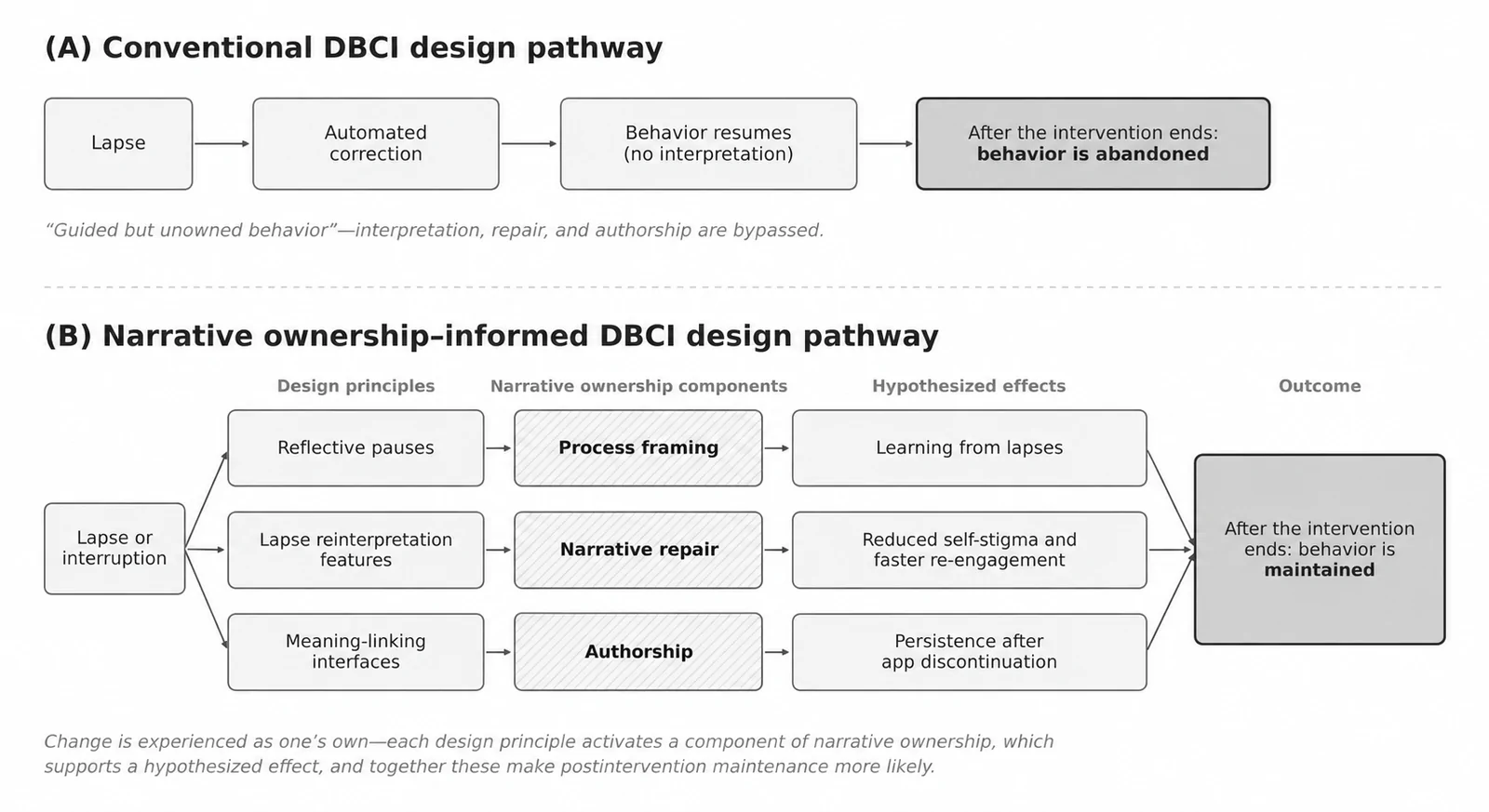
Comparison of the 2 design pathways: (A) conventional digital behavior change intervention (DBCI) design pathway vs (B) narrative ownership–informed DBCI design pathway.

## Testability and Limitations

### Testable Propositions

The design principles yielded the following 3 testable propositions. First, a DBCI that incorporates reflective pauses following detected lapses will produce higher rates of behavioral maintenance at 3 and 6 months after intervention than a functionally equivalent DBCI that delivers immediate algorithmic correction. The hypothesized mechanism is that reflective pauses preserve process framing. Second, users of a DBCI that includes lapse reinterpretation features will report lower self-stigma following lapses and faster re-engagement with behavioral targets than users of a DBCI that uses standard notification-based redirection. The hypothesized mechanism is that narrative repair reduces the abstinence violation effect [28]. Finally, users of a DBCI with meaning-linking interfaces will demonstrate higher behavioral persistence after discontinuing app use than users of a DBCI with standard gamification. The hypothesized mechanism is that meaning-linking cultivates authorship that persists after the external scaffolding is removed. Testing these propositions requires explicit outcome definitions. Here, behavioral maintenance refers to meeting the target behavior at 3 and 6 months after the intervention ends; re-engagement refers to the time from a lapse to resumption of the target behavior; and authorship refers to the degree to which users attribute the change to themselves rather than to the app and, beyond attribution, can describe it in their own words and connect it to goals that matter to them personally or relationally; it is operationalized separately from autonomous motivation and self-efficacy.

These propositions can be tested independently or using factorial designs that evaluate the individual and combined effects of each component [57]. Empirical progress also requires the development of measures of narrative ownership (with subscales for process framing, narrative repair, and authorship)—for example, process framing items might assess whether users view a lapse as part of a longer change trajectory, and authorship items might assess whether the change feels “my own” rather than “caused by the app,” whether users can describe it in their own words, and whether they connect it to goals that matter to them personally or relationally—that demonstrate discriminant validity relative to self-efficacy [40], autonomous motivation [15], and engagement [18]. Existing instruments for narrative identity [58] and the recently developed Narrative Identity Self-Evaluation Scale [59] may provide useful starting points.

### Limitations

This viewpoint has some limitations that warrant further discussion. First, the framework is entirely conceptual, and the design principles have not been empirically tested. Second, reflective pauses and lapse reinterpretation prompts require cognitive effort and may reduce engagement among users with low motivation or under high cognitive load [51]. For some users—including those experiencing depression, anxiety, low health literacy, or socioeconomic stress—repeated reflection after lapses may heighten guilt or rumination rather than promote learning, so reflective prompts should be tailored to the user and remain suppressible. Whether these features can be designed to be brief, adaptive, and optional, without diluting their reflective functions, remains an empirical question. Third, the framework was developed primarily based on chronic relapse-prone behavioral domains. However, its applicability to acute or 1-time behavior changes may be limited.

Fourth, the expression of narrative ownership varies across cultural contexts; the emphasis on individual authorship may reflect individualistic Western assumptions [39], and in collectivist contexts ownership may instead be familial or communal, so cross-cultural validation is essential for the framework’s generalizability [60]. Practical barriers also warrant consideration: reflective and meaning-linking features compete for limited screen space, may add to user fatigue, and are difficult to integrate into commercial apps built for frictionless engagement. Because narrative data are sensitive—spanning personal values, relationships, and illness experiences—their collection further raises privacy and data governance obligations that interventions must address [61]. At a minimum, such interventions should collect no more narrative data than necessary, process reflective content on the device where this is possible, encrypt what is stored, seek explicit and specific consent, and give users a straightforward way to review, export, or delete their own entries. Finally, the framework does not address the structural and socioeconomic determinants that constrain individuals’ capacity to engage in behavior change, regardless of narrative ownership [60]. It is intended to complement, not replace, the attention paid to these factors.

## Conclusions

This viewpoint proposed a sustainability paradox at the heart of contemporary DBCI design. The features that make interventions effective in the short term may simultaneously displace the experiential processes through which behavior change becomes personally owned. We proposed narrative ownership, comprising process framing, narrative repair, and authorship, as a framework for understanding this paradox and translated it into 3 design principles that can be embedded in digital interventions. Thus, the core argument is straightforward. A person may meet their daily step target for months if their smartwatch repeatedly prompts them to move. However, once the device is removed or notifications stop, the walking routine often disappears. Guided but unowned behavior is vulnerable to abandonment. If the goal of digital health interventions is to support lasting change, that is, change that persists in the user’s life after the app is set aside, then the design question must expand beyond “How do we get users to do the right thing?” to include “How do we help users experience the change as their own?” Narrative ownership offers a framework for pursuing this question that positions the user not merely as the recipient of guidance but also as the author of change.

## Abbreviations

BCT: behavior change technique
DBCI: digital behavior change intervention
JITAI: just-in-time adaptive intervention
mHealth: mobile health
SDT: self-determination theory
